# Functional balance training in people with Parkinson’s disease: a protocol of balanceHOME randomized control trial with crossover

**DOI:** 10.3389/fnagi.2023.1137360

**Published:** 2023-04-26

**Authors:** Sara Monleón Guinot, Constanza San Martín Valenzuela, Vivina Aranda Asensi, Concepción de Salazar Antón, Manuel Villanueva Navarro, Jose M. Tomás

**Affiliations:** ^1^Department of Methodology for the Behavioral Sciences, Faculty of Psychology, University of Valencia, Valencia, Spain; ^2^Unit of Personal Autonomy, Dependency, and Mental Disorders Assessment, INCLIVA Biomedical Research Institute, Valencia, Spain; ^3^Departament of Physiotherapy, Faculty of Physiotherapy, University of Valencia, Valencia, Spain; ^4^Centro de Investigación Biomédica en Red de Salud Mental (CIBERSAM), Instituto de Salud Carlos III, Madrid, Spain; ^5^Asociación Parkinson Valencia Neurorehabilitation Center, Valencia, Spain

**Keywords:** Parkinson’s disease, functional balance, physiotherapy, home, domiciliary, protocol, Randomizad controlled trial, crossover

## Abstract

**Introduction:**

Balance disturbances in Parkinson’s Disease (PD) are usually assessed in a single-task as well as standard balance physiotherapy is carried out in isolated environments. Conversely, daily activities are developed in highly challenging environments. Although functional balance training (FBT) is included in the latest protocols, several methodological issues have not yet been considered. In the proposed single-blinded randomized control trial with crossover (NCT04963894), the aims are (1) to quantify the effects achieved by domiciliary FBT (balanceHOME program) in participants with and without cognitive impairment, and (2) to compare them with the effects of a passive-control period and a conventional face-to-face physiotherapy program for PD.

**Methods:**

The initial recruitment was estimated at 112 people with idiopathic PD. Two-thirds of the participants will be randomized to one of the two groups to make the crossover. In contrast, the other third will do a face-to-face group program only. The balanceHOME protocol consists of challenging balance exercises incorporated into functional daily tasks, developed in-home and conducted two times per week for 60-min over an 8-weeks period. The primary strategy will consist of splitting functional tasks of daily life into static and dynamic balance components, besides standardized facilitate and disturbing strategies to execution of each exercise. Biomechanics and clinical performance of balance and gait, perception of quality of life, cognitive and mental functioning, and severity of PD will be measured at baseline (T0), post-8 weeks training (T1), and follow-up (T2).

**Results:**

The primary outcome of the study will be the center of pressure sway area. The secondary outcomes consist of biomechanics and clinical variables related to static and dynamic balance. Outcomes from biomechanical of gait, quality of life, cognitive and mental state, and severity of PD, represent the tertiary outcomes.

**Discussion:**

The balanceHOME program standardizes the FBT in demanding and daily environments for people with PD who prefer individualized treatment from home. This is the first time that the effects of group versus individual balance rehabilitation have been compared in people with and without cognitive impairment and evaluated in complex environments. This still-to-be-finished study will open the possibility of new strategies according to changes in post-pandemic therapeutic approaches.

## 1. Introduction

Parkinson’s disease (PD) is a progressive neurodegenerative disease characterized by motor and non-motor symptoms, including bradykinesia, resting tremor, muscle rigidity, flexion posture, walking and balance impairment, autonomic dysfunctions, sleep disorders, cognitive disturbances, and behavioral alterations (Poewe et al., 2017). The balance disturbances in PD consist of biomechanical restrictions on support base width, postural misalignment, muscle weakness at the ankle and hip for standing, limits of stability (LoS) restriction (Kara et al., 2012; Soke et al., 2019) perception of verticality loss, absence or delay of anticipatory postural adjustments, impaired sensorimotor integration, poor control of the center of pressure (CoP) (Carpinella et al., 2017; Santos et al., 2017), loss of dynamic balance during walking (Wong-Yu and Mak, 2015a; Klamroth et al., 2016; Fil-Balkan et al., 2018; Giardini et al., 2018), and gait speed decrease (Conradsson et al., 2015; Giardini et al., 2018; Wallén et al., 2018). As a result of these disturbances, the risk to fall forward (propulsion) or backward (retropulsion) appears, which increases with the PD progression (Allen et al., 2011; Park et al., 2015; Yitayeh and Teshome, 2016; Hubble et al., 2018). Consequently, these events affect a wide range of daily life activities and participation (World Health Organization (WHO), 2001), impacting functional independence and the quality of life of people with PD (da Capato et al., 2015; Park et al., 2015; Yitayeh and Teshome, 2016).

Balance alterations in PD are usually assessed in a single task. The most common tests are based on static and dynamic balance, where the patient controls the CoP in a static position (e.g., standing) (Negrini et al., 2017; Giardini et al., 2018; Soke et al., 2019) or during motion in direction to the different LoS (Kara et al., 2012; Carpinella et al., 2017; Soke et al., 2019). To this assessment methodology, it is common to add the blockade of the different systems that participate in balance regulation, either by closing the eyes or placing foam under the feet. Similarly, standard balance physiotherapy is carried out in isolated environments with exercises focused on single tasks or similar scenarios to those of the evaluation (Giardini et al., 2018). Conversely, daily activities are developed in highly challenging environments, which have been addressed in previous publications with Wii balance games (Yen et al., 2011; Zalecki et al., 2013; Carpinella et al., 2017; Negrini et al., 2017; Feng et al., 2019; Yuan et al., 2020), dual-task exercises within daily activities (Conradsson et al., 2015; Wong-Yu and Mak, 2015a, 2015b; Atterbury and Welman, 2017; Perumal et al., 2017; Santos et al., 2017; Wallén et al., 2018; Leavy et al., 2020), and single daily activities training (Smania et al., 2010; Kara et al., 2012b) including postural transferences (Millage et al., 2017; Soke et al., 2019). Although functional balance training (FBT) is included in the latest protocols, which consists of daily activities with feasible settings for older adults with PD, there are several methodological issues that have not yet been considered. One of these is the assessment methodology of the previous studies and the objectivity of the measurements, using in most cases, scales, and clinical tests only (Conradsson et al., 2015; Wong-Yu and Mak, 2015a,b; Atterbury and Welman, 2017; Perumal et al., 2017; Santos et al., 2017; Wallén et al., 2018; Leavy et al., 2020) instead of integrating biomechanical parameters of balance in different measurement conditions with a follow-up period after finishing the studied intervention (Kara et al., 2012b; Conradsson et al., 2015; Atterbury and Welman, 2017; Perumal et al., 2017; Santos et al., 2017; Soke et al., 2019; Leavy et al., 2020). Furthermore, the authors who inform significant improvement in balance biomechanics (Kara et al., 2012; Santos et al., 2017; Soke et al., 2019) do so in patients with normal cognition status and use habitual locations like a physiotherapy gym or hospital facilities. To respond to the challenges that emerged since the COVID-19 pandemic, such as the demand for rehabilitation programs that can be followed from home, the effect of domiciliary FBT and the long-term impact with adequate follow-up need still be determined, as well as the effects in people with PD in advanced stages where cognition is altered. For the foregoing, the aims of this study are (1) to quantify the effects achieved by domiciliary FBT on the biomechanics of balance, gait, perception of quality of life, cognitive and mental performance, and severity of PD in participants with and without cognitive impairment and (2) to compare them with the effects of a passive-control period and a conventional face-to-face physiotherapy program for PD. We hypothesized that balance home training based on functional exercises has a greater effect than the traditional physiotherapy group. If this hypothesis is confirmed, it will open the possibility of new rehabilitation strategies according to changes in post-pandemic therapeutic approaches, reducing morbidity, and saving costs to the healthcare system.

## 2. Materials and analysis

### 2.1. Study design

The balanceHOME trial is designed as a randomized, controlled, and blind evaluator trial with a crossover intervention group. To write this protocol was used as a guide the Standard Protocol Items for Randomized Trials (SPIRIT) guidelines (Chan et al., 2013) and the Consolidated Standards of Reporting Trials statement for randomized controlled trials (Moher et al., 2010). The SPIRIT guidelines can be consulted in Supplementary material 1. Once the study was designed, it was registered on ClinicalTrials.gov (NCT04963894) on 15 July 2021 with the approval information of the Human Research Ethics Committee of the Experimental Research Ethics Commission of the University of Valencia obtained (Supplementary material 2).

**Figure 1:**
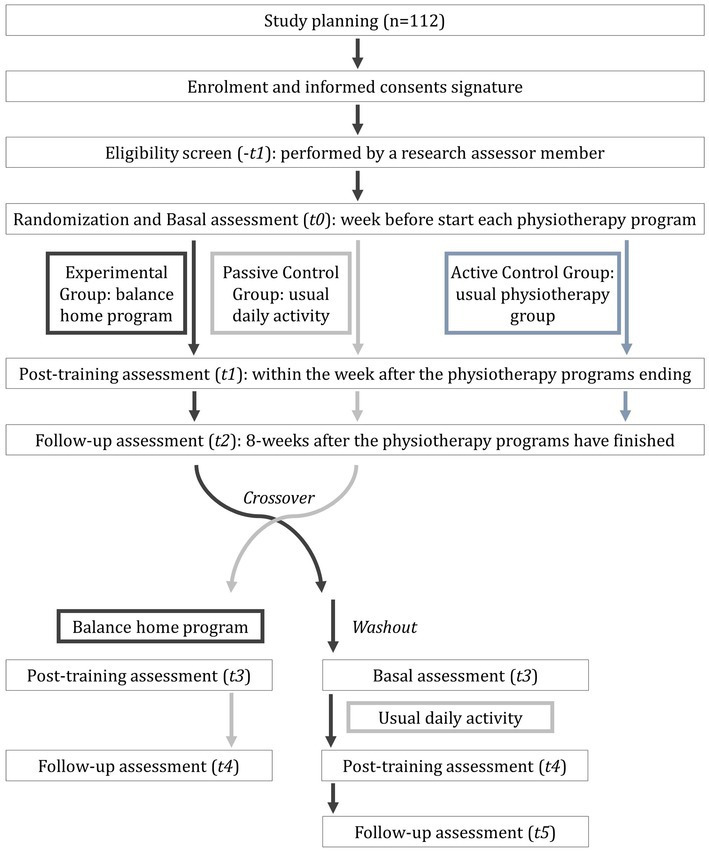
Schedule of enrolment, interventions, and assessments of balanceHOME randomized control trial study.

The balanceHOME will be started with a baseline assessment session (T0) and followed by random allocation to one of two study groups: the experimental group (EG) or the passive control group (PCG). Additionally, a third group of participants with PD who habitually developed a conventional group physiotherapy program (Active Control Group, ACG) will be included in the evaluation process. At the end of the interventions (8 weeks), a post-training evaluation (T1) will be carried out, and subsequently, a follow-up assessment (T2) 8 weeks after the physiotherapy programs have finished. At this point, patients from PCG will switch with EG to receive the functional balance home program. The EG will follow a 4-month washout period (Serrao et al., 2019) before continuing as passive control participants (see Figure 1). The crossover design allows to control the intra-individual changes (due to personal characteristics and disease presentation), offer all the research benefits to the participants, and maximize the sample size it can achieve.

### 2.2. Participants, interventions, and outcomes

#### 2.2.1. Setting and eligibility criteria

The recruitment of participants, the eligibility criteria assessment, obtaining informed consent (Supplementary material 3), and the intervention of the ACG will be carried out in the facilities of the neurorehabilitation center participant in the study. On the other hand, the pre-intervention, post-intervention, and 8-week follow-up evaluations will be carried out at the University of Valencia, while the interventions of the EG will be carried out at each participant’s home.

The recruitment and eligibility criteria assessments will be carried out by two different physiotherapists, who will verify the following inclusion criteria: (i) idiopathic PD; (ii) Hoehn & Yahr stage I-IV; (iii) independent gait with or without technical assistance; and (iv) stable parkinsonian medication for at least a month before the study star. On the contrary, the exclusion criteria will be as follows: (i) comorbidities that affect balance or walking; (ii) other neurological pathologies besides PD; (iii) chronic diseases not medically controlled; and (iv) participation in another physiotherapy program or sport activity during the intervention period or in the month before starting the study. Patient enrolment was started in August 2021 and is expected to be completed in August 2023. All patients are expected to have completed baseline testing in September 2023.

#### 2.2.2. Interventions

In total, two physiotherapy programs have been designed (balanceHOME and standard physiotherapy), both conducted two non-consecutive days per week, for 60 min each session over an 8-week period (Wong-Yu and Mak, 2015a,b; Atterbury and Welman, 2017; Santos et al., 2017; Soke et al., 2019), and each program will be conducted by different professionals. The sessions of both programs also have the same structure: warm-up (10′), central phase (45′), and cool-down (5′). The balanceHOME program will be performed for the EG, while the ACG will perform the standard physiotherapy program with the conventional goals and exercises for PD. This standard program will be developed in groups of six participants and conducted by a physical therapist and a rehabilitation assistant. The objectives included in the conventional program are (San Martín Valenzuela et al., 2020b): (i) cardiovascular training; (ii) upper and lower body strengthening; (iii) trunk strengthening and control; (iv) somatosensory balance; (v) dynamic balance; (vi) weight transfer and reaching; (vii) walking; (viii) fine motor skills; and (ix) stretching and relaxation.

On the other hand, the balanceHOME program consists of challenging balance exercises incorporated into functional daily tasks developed in-home and conducted by a different physiotherapist from the standard intervention. Additionally, whenever possible, the presence of a family member or caregiver in each session will be requested. The primary strategy will consist of splitting functional tasks of daily life (see Table 1) into static and dynamic balance components to facilitate an individualized progression in a functional context. Each exercise in the sequence will be developed in 30–60″ and will be repeated until the patient performs the necessary support reactions to avoid falling or has no imbalances to complete the exercise before continuing to the next level of the sequence. The following adaptations will be used to facilitate the execution of exercises from Table 1: (i) wide base of support; (ii) stable surfaces and flat ground; (iii) use of footwear; and (iv) hand-holding points. Conversely, the following balance disturbing strategies will be used before jumping into the next level of a sequence: (i) reduced support base; (ii) sloping floor and unstable surface; (iii) destabilizing forces (da Capato et al., 2015; Plate et al., 2016) by external imbalances; (iv) LoS exploration; (v) barefoot; and (vi) dual-task (cognitive and motor). Foam rubber, balls, and steps, as well as elements or set-ups from the home of each participant, will be used in each session. Regarding the warm-up phase, exercises related to breathing (Pitts et al., 2009; Reyes et al., 2013; van de Wetering-van Dongen et al., 2020), shoulder and pelvic girdle dissociation, joint mobility, postural changes (Stożek et al., 2016), muscle strengthening (Meyer et al., 1965; Kanegusuku et al., 2021), upper and lower limb mobility, and gait (Tomlinson et al., 2013; Tambosco et al., 2014) will be included. Finally, the cool-down phase will be focused on muscle stretching of the upper and lower limbs, trunk, head, and neck.

**Table 1:** Functional tasks and balance components from the balanceHOME program.

Functional task	*Static balance component*	*Dynamic balance component*
1. Reaching objects in sitting position	Lateral reaching outside the body midline. Frontal reaching outside the body midline.	Reaches outside the body midline while moving to standing position.
2. Looking in different directions during standing position	Head movement to different directions. To visualize objects in lateral visual field. To visualize objects in anterior visual field.	Talking to another person and walking. Avoid objects in the field of vision while walking.
3. Dressing during standing position	Fasten shirt buttons while standing. Tie your shoes while squatting.	Simulate pulling up your pants during the change from sitting to standing.
4. Manipulation above 90° of shoulder flexion during standing position	Cleaning a wall mirror while standing Reach for high shelf items.	Running and pulling back a curtain doing lateral gait.
5. Manipulation below 90° of shoulder flexion during standing position	To clean a piece of furniture. Watering plants.	Look for an object inside a bag while walking.
6. Ball throws and receptions during standing position	To throw an object at another person while standing. To catch an object in the air while standing.	To throw an object at another person while walking. To catch an object in the air the change from sitting to standing.
7. From seated to standing	Hold the semi squat position without upper limb support.	Go from sitting to standing position from a low seat.
8. From standing to squat to pick up objects from the ground	Pick up an object from the ground by flexing the trunk.	To pick up an object from the floor, with the semi-kneeling position.
9. From sitting on the ground to standing	Quadruped position alternating support between lower and upper limbs and change to three-points of support.	Moving from sitting on the floor to a semi-kneeling position and standing.
10. Steps	One foot on a step. To reach an object above 90° of shoulder flexion while on the steps.	To go up and down the steps. About passing the steps.
11. Stairs	One leg support with the freedom leg in different positions and heights.	To go up and down the stairs in single and dual-task.
12. Gait	Standing on irregular support base. Standing on narrow support base.	Gait with the support base altered. Walking through circuits with obstacles.
13. Manipulation objects during gait	Standing carrying an object of different sizes and different distances from the body.	Gait carrying an object of different sizes and different distances from the body.
14. Walking outside	Standing on the street with external cognitive and motor disturbances.	Gait on the street with external cognitive and motor disturbances.
15. Manipulation objects during walking outside	Standing outside and carrying an object of different sizes and different distances from the body.	Walking outside and carrying an object of different sizes and different distances from the body.
16. Coordination	To learn a sequence of movements with the arms while standing.	To learn a sequence of movements with the arms and legs.

#### 2.2.3. Outcomes and participant timeline

**Figure 2:**
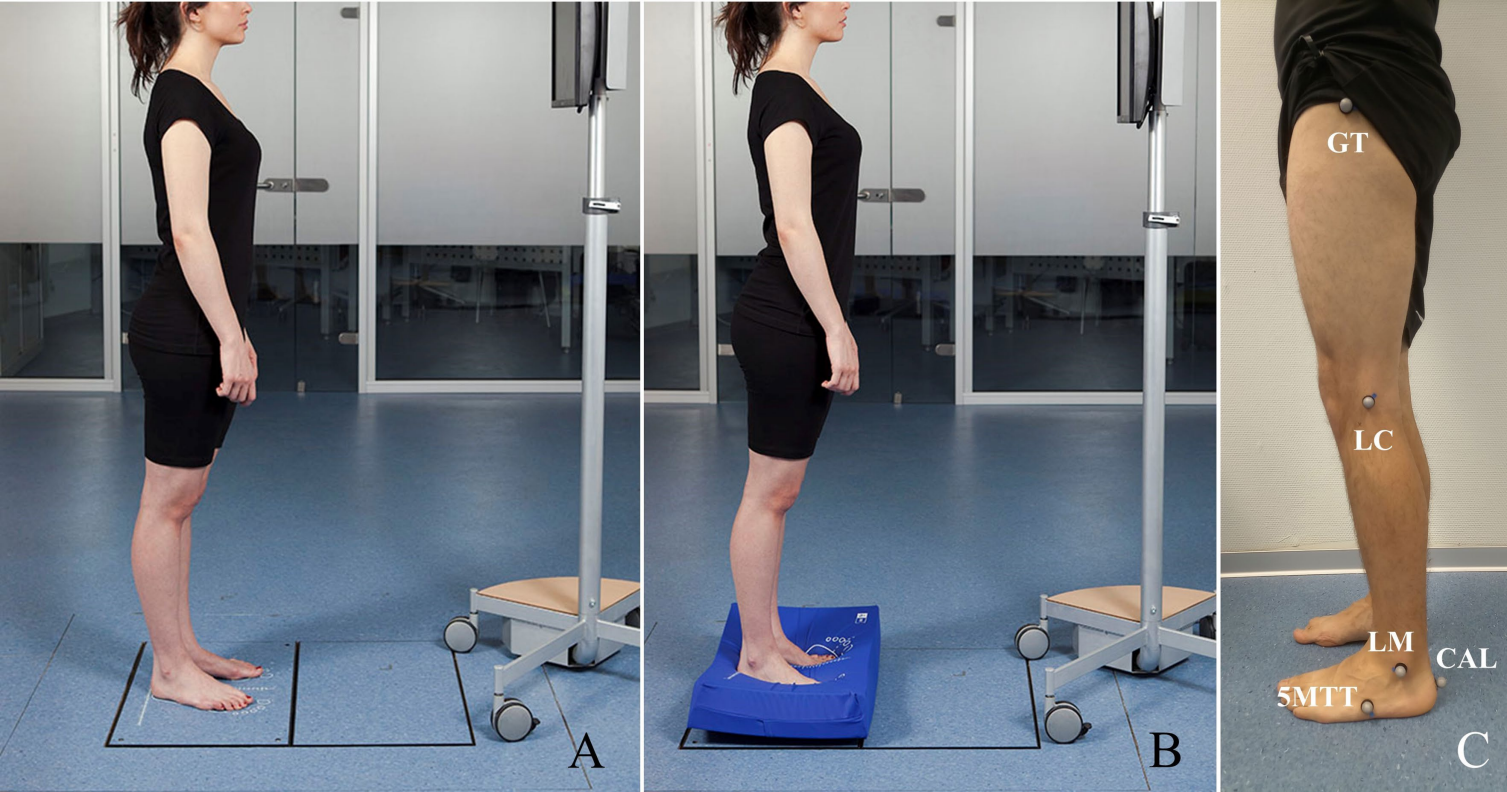
Static balance test. **(A)** Protocol for the Romberg test without a foam rubber and for the visual, verbal, lower and upper motor static functional test. **(B)** Protocol for the Romberg test with a foam rubber under the feet. **(C)** Gait model composed by tuberosity of fifth metatarsal (5MTT), posterior surface of calcaneus (CAL), greater trochanter of the femur (GT), lateral condyle of the knee (LC), and lateral malleolus of the ankle (LM).

First, during the enrolment assessment, a standardized neurological clinical interview for Parkinson’s Disease (Supplementary material 4) will be performed. This interview includes questions related to habits, disease start, education, and working life. At the same time, weight, height, and body mass index will be measured with a wall stadiometer and a TANITA SC-240MA scale. The length of the lower limbs will also be measured during standing position with a tape measure from the anterior superior iliac spine and the medial malleolus. Asymmetries between the lower extremities greater than 1 cm will be considered within the first exclusion criteria (comorbidities that affect balance or gait). As mentioned above, each phase of the study has three evaluation moments: baseline (T0), post-8-week-training (T1), and follow-up (T2) at 8 weeks after the physiotherapy programs have finished. Assessments will be performed by a different physiotherapist from recruitment and treatment besides a neuropsychologist, both blinded to the treatment assignment. The participants will be evaluated in an ON-medication state (1 h after the dopaminergic dose) on the schedule described in Figure 1. In each measurement session, the severity of the disease (Hoehn and Yahr, 1967; Fahn et al., 1987), cognitive and mental status (Hamilton, 1959, 1960; Mahieux et al., 1995; Dubois et al., 2000; Marinus et al., 2003), perception on quality of life (Peto et al., 1995; Powell and Myers, 1995; Sandín et al., 2020), and balance and gait performance will be registered through clinical tests (Tinetti, 1986; Giladi et al., 2000; Kegelmeyer et al., 2007; Franchignoni et al., 2010) as well biomechanics instruments. Table 2 shows the scales and clinical tests that will be used to evaluate the participants. For balance biomechanics, assessment will be used a dynamometric platform (Dinascan/IBV Biomechanics Institute of Valencia, Valencia, Spain) and the NedSVE®/IBV software (version 5.3.0.1, Biomechanics Institute of Valencia, Valencia, Spain). Balance will be assessed through the Romberg tests under four static conditions with increasing difficulty (see Figures 2A,B): (i) eyes open; (ii) eyes closed; (iii) eyes open on a foam rubber; and (iv) eyes closed eyes on the foam rubber (thickness of 9 cm, 56.7 kg/m3 of density, and resistance of 25%) (Escamilla-Martínez et al., 2021). Participants were asked to find the most stable position while barefoot with their arms relaxed on either side of the body, with their heels together and toes apart at a 30° angle. Additionally, four static functional balance tests will be assessed, using the Romberg test protocol with their eyes open: (i) to see the time on an analogy clock (visual); (ii) to answer a simple question (verbal); (iii) to pour the contents of one glass into another (lower motor); and (iv) to simulate comb the hair (upper motor). The instructions for each task were standardized to ensure uniform execution of the gesture. Participants will be asked to remain as still as possible with their heads in a neutral position and their gaze forward, except for the lower motor test where they will be allowed to observe their hands. Each test will last for 30 s and will be recorded two times. Finally, dynamic balance will be assessed with stability limits and rhythmic weight shift tests on the platform (Balaguer García et al., 2012). In the stability limits test, participants from the Romberg position will have to move a cursor (which reflected the position of their CoP) toward eight targets (front, front-right, right, rear-right, rear, rear-left, left, and front left) visualized on a computer monitor placed in front at their eye level. Once the target is reached, the subject will have to maintain the posture with the CoP displaced at the limit of stability until the end of the 8 s of each test (Balaguer García et al., 2012). On the other hand, in the rhythmic weight shifts tests, subjects will have to move their CoP to the direction and velocity of a moving target visualized on the computer monitor (Balaguer García et al., 2012). Each dynamic test will be registered two times. As a result, the primary outcome of the study will be the CoP sway area, which is defined as the surface over which the CoP moves during the measurement and is a valid indicator of detecting postural control changes in dual-task conditions (Campolettano et al., 2020; Morenilla et al., 2020). The secondary outcomes are shown in Table 2 and consist of biomechanics and clinical variables related to static and dynamic balance.

**Table 2:** Summary of outcomes, measurement instruments, and assessment times.

Domain / Outcomes	Measurement instrument	Study period			
		Enroll (−t1)	Basal (t0)	Post (t1)	Follow (t2)
Enrollment					
Eligibility screen	Participation criteria	x			
Informed consent	Supplementary material 3	x			
Personal information	SNCI for PD (Supplementary material 4)				
Habits and working life					
Anthropometric data (weigh, height, body mass index, and lower limb length)	Wall stadiometer, TANITA SC-240MA scale, and tape measure	x			
Parkinson’s disease information					
Disease start and initial sign	SNCI for PD	x			
Parkinson’s disease severity	H&Y; UPDRS-III	–	x	x	x
Cognitive and mental status					
General cognitive state	MMP; SCOPA-COG	–	x	x	x
Executive cognitive function	FAB	–	x	x	x
Anxiety	HS for anxiety				
Depression	HS for depression	–	x	x	x
Balance and gait clinical performance					
Balance	MBT; TMT balance	–	x	x	x
Gait	TMT gait	–	x	x	x
Freezing of gait	FOG-Q	–	x	x	x
Quality of life					
Impact on life due COVID pandemic	CPI-Q		x	–	–
Perception of QoL due to Parkinson’s disease	PDQ-39		x	x	x
Confidence on self-balance	ABC scale				
Balance biomechanical performance					
*Static balance: Romberg and Functional test*					
ML and AP OP displacement (mm)	Dynamometric platform	–	x	x	x
Total CoP displacement (mm)		–	x	x	x
CoP displacement angle (°)		–	x	x	x
Mean CoP velocity (ms^−1^)		–	x	x	x
ML and AP CoP dispersion (mm)		–	x	x	x
CoP swept area (mm^2^)		–	x	x	x
*Dynamic balance: stability limits test*					
Reaction time (*s*)	Dynamometric platform	–	x	x	x
Maximum CoP displacement (%)		–	x	x	x
Success (%)		–	x	x	x
Directional control (%)		–	x	x	x
*Dynamic balance: rhythmic and directional control test*					
Directional adjustment (%)	Dynamometric platform	–	x	x	x
Perpendicular adjustment (%)		–	x	x	x
Gait biomechanical performance					
Speed (ms^−1^) and speed variability	3D photogrammetry Dynamometric platform	–	x	x	x
Stride length (m) and time (*s)*		–	x	x	x
Step length (m) and width (*m*)		–	x	x	x
Cadence (steps/min)		–	x	x	x
Double support time (%)		–	x	x	x
Stance and swing phase time (*s*)		–	x	x	x
Maximum ankle dorsiflexion during swing (°)		–	x	x	x
Maximum knee flexion during swing (°)		–	x	x	x
Maximum hip extension during stance (°)		–	x	x	x
Maximum hip flexion during swing (°)		–	x	x	x
Range of motion of lower limb joint (°)		–	x	x	x
CoP medial-lateral displacement (mm)		–	x	x	x
Mediolateral force amplitude (*N*)		–	x	x	x

SNCI for PD, Standardized neurological clinical interview for Parkinson’s Disease; H&Y, (Hoehn and Yahr, 1967); UPDR-III, Unified Parkinson’s Disease Rating Scale part III (Fahn et al., 1987); MMP, Mini-Mental Parkinson (Mahieux et al., 1995); SCOPA-COG, Scale for Outcomes in Parkinson’s disease-Cognition (Marinus et al., 2003); FAB, Frontal Assessment Battery (Dubois et al., 2000); HS, Hamilton scale (Hamilton, 1959, 1960); MBT, Mini BESTest (Franchignoni et al., 2010); TMT, Tinetti Mobility Test (Tinetti, 1986); FOG-Q, Freezing of gait questionnaire (Giladi et al., 2000); CPI-Q, Coronavirus psychological impact questionnaire (Sandín et al., 2020); PDQ-39, Parkinson’s Disease Questionnaire (Peto et al., 1995); ABC scale, Activities-specific Balance Confidence Scale (Powell and Myers, 1995). CoP, center of pressure.

In addition to balance assessment, the gait of participants will also be measured in a 10 m-long, straight and flat walkway by NedAMH+®/IBV software (v1.1.1, Institute of Biomechanics of Valencia, Spain) which uses two red-light photocells to measure gait speed besides a 3D photogrammetry system with 12 smart-cams (Kinescan®/IBV, version 5.3.0.1, Biomechanics Institute of Valencia, Valencia, Spain), and the dynamometric platform mentioned above. The biomechanical gait model aims to measure movement in the sagittal plane and is made up of 10 landmarks (5 on each leg), located at the anatomical points in Figure 2C. A total of 10 walking repetitions will be analyzed, of which five corresponded to the force data of the right footprint and the other five to the left footprint. Participants will be allowed to walk along the corridor a few times before recording their gait (San Martín Valenzuela et al., 2020a,b). The outcomes from the gait biomechanical assessment are shown in Table 2, and they represent the tertiary outcomes of this study along with those outcomes related to the quality of life, cognitive and mental state, and severity of PD.

#### 2.2.4. Sample size and recruitment

The software G*Power v.3.1 (Faul et al., 2007) has been used to determine the necessary sample size to detect a change in the primary outcome CoP sway area and differences between the groups (EG, PCG, ACG), with a small-medium effect (*f* = 0.15), a statistical significance of 5% at the two-tailed level, and a power of 95%. As a result of the above, 93 people diagnosed with PD will be recruited. If 20% of possible dropouts are also considered during the study, the initial recruitment should be 112 people. The sampling process will consist in the consecutive non-probabilistic method, in which the selection of individuals is carried out based on the fulfillment of participation criteria. The recruitment of participants began on 1 July 2021 and we estimate its completion on 31 July 2023. The participant recruitment procedure consists of three steps. First, the neurorehabilitation center has selected the possible candidates for the study based on personal and clinical characteristics, asks them by email the authorization to send information about the study, and contacted them using their telephone number. Then, the principal investigators called potential candidates to resolve possible doubts and confirm their participation. Finally, a physiotherapist will assess the selection criteria for all the volunteers and will provide informed consent for their signatures. The recruitment process will be repeated to complete the number of participants estimated for the study, extending participation to a new clinical center or public hospital if necessary.

### 2.3. Assignment of interventions and blinding

An external investigator will perform the randomization process (1:1). Stratified randomization will be carried out according to PD severity and general cognitive state and assigned to one of the two groups for crossover, EG or PCG. A matched-pairs design will be created in which participants were allocated to these two groups taking into consideration the outcomes, i.e., Hoehn and Yahr state (I-II-III-IV) and Mini Mental Parkinson Test score (≥ 25, normal cognitive function or ≤ 24, altered cognitive function) at T0. The principal investigators will be responsible for the management of participants’ appointments after being assigned by telephone. On the other hand, the physiotherapist evaluator will be blinded to the assignment of the groups. For this reason, it will be explained to the participants that they do not reveal information about their intervention during the assessment sessions. Due to the nature of interventions, the patients and the physiotherapist who develop the rehabilitation cannot be blind to the treatment to be performed, but the hypothesis of the study will be hidden. Additionally, after the consent signature, participants will be assigned a numeric code to hide the group of intervention.

### 2.4. Data collection, management, and statistical analysis

Both evaluators (physiotherapist and neuropsychologist) will be responsible for exporting and filling in a database with variables from the scales and tests mentioned in Table 2 according to the code assigned to each patient to ensure his or her anonymity. To protect confidentiality during the study, the personal information of participants will be located separately from the main dataset on a local computer. The raw dataset will be maintained for 10 years after the completion of the trial with indefinite restricted access due to sensitive data. After the publication of the results trial, a fully anonymized patient-level dataset will be made publicly available on the registration trial website (clinicaltrials.gov). For participation in this study, no participant will receive any kind of incentives or compensation.

All analyses will be evaluated by intention-to-treat principles in terms of assignment to treatment and used a level of significance of 0.05. There are no interim analyses planned for this study. We will use the statistical software SPSS Statistics version 24.0 (IBM Corporation) for all the statistical analyses. Categorical variables were presented using frequency and percentage, and continuous variables as mean with standard deviation if they follow a normal distribution. To test for possible carryover effects of the experimental group, the sum of the values measured in the two periods for each subject will be calculated and compared across the two randomized groups (EG and PCG) using a test for independent samples (Serrao et al., 2019). There should be no difference if there is no sequence (balanceHOME program—usual daily activity or usual daily activity—balanceHOME program) effect.

To answer to the main aim of the study, a two-factor mixed multivariate analysis of variance (MANOVA) test was conducted to analyze the effects of within-subject factors (assessment times) and the between-subject factor (group) on the outcomes registered. The Bonferroni adjustment was used for *post-hoc* comparisons, and differences were declared statistically significant if the value of *p* was less than 0.05. Differences between groups for demographic outcomes were verified with a univariate analysis of variance (ANOVA) between-subjects test. Furthermore, to test for sex differences between groups, a chi-square analysis was used (San Martín Valenzuela et al., 2020b).

### 2.5. Monitoring

No external professionals will be contact for data monitoring procedures. Rather, internal data monitoring is coordinated by a Data Monitoring Committee. For this purpose, one member of each institution of the study integrates the Committee. The monitoring will be independent of the evaluation appointments of this study and will be carried out by telephone monthly to all participants to record the following: (1) changes on Parkinsonian or other medication, (2) falls/week or other adverse events, (3) changes on other therapies (psychology or speech therapy) and neurology appointments, and (4) changes in physical activity outside the study. The Data Monitoring Committee will decide whether trial participation should be discontinued based on the reports from monitoring. The monitoring data will be anonymized and published once the study is over on the trial registration website (clinicaltrials.gov).

## 3. Discussion

Traditionally, balance rehabilitation in people with PD was based on single-task exercises which poses unrealistic scenarios contrary to the complexity of daily life activities where people control multiple systems at the same time in response to multiple stimuli and demands. For this reason, the aim of this study is to quantify the effects of functional balance home training on the biomechanics of balance, gait, general physical status, cognitive performance, and quality of life. Previous authors have considered that including functional tasks in rehabilitation could be more beneficial for people with PD as it guides them back to their everyday lives. In this line, some authors have studied this issue through training with Wii technology (Yen et al., 2011; Zalecki et al., 2013; Carpinella et al., 2017; Negrini et al., 2017; Feng et al., 2019; Yuan et al., 2020), and others have designed easily reproducible training with daily task. However, of these latter authors, only a few have included biomechanical objective outcomes in the evaluation that allow the effects of interventions to be accurately quantified (Smania et al., 2010; Kara et al., 2012; Santos et al., 2017; Soke et al., 2019). The improvements reported by the authors reach 57% on postural stability (Soke et al., 2019), 12,54% (Kara et al., 2012) and 25% (Soke et al., 2019) on LoS exploration, 36,42% (Santos et al., 2017) and 42% (Smania et al., 2010) on CoP sway area, and 40,48% on CoP sway velocity during the Romberg test (Kara et al., 2012). Although the balance changes reported by the studies are significant, the samples of these trials are small and do not include the effectiveness results of patients with cognitive impairment. This aspect is considered in this protocol since epidemiologic studies indicate that the cumulative prevalence of Parkinson’s disease dementia in 8 years is as high as 78.2% and approximately 40% of PD subjects at an earlier stage have a co-existing mild cognitive impairment, boosting the risk of converting to PDD (Fang et al., 2020). This is relevant since the improvement capacity of patients with some cognitive deficit with respect to those with normal cognition is not known even though they have already described the relationship between balance disturbances and cognitive impairment (Saricaoglu et al., 2021). In this sense, home rehabilitation offers an easy alternative for people with PD and their families and can provide greater rehabilitation opportunities and independence for people who must remain at home.

Another relevant aspect is that this protocol aims to solve is the inclusion of dual tasks in the evaluation of balance. Biomechanical balance is usually evaluated as single-tasks on the dynamometric platform (Smania et al., 2010; Kara et al., 2012; Santos et al., 2017; Soke et al., 2019). Complementing the static balance test with dual tasks would provide results of balance performance in real-world situations. Previously, the impact of dual tasks on the gait of trained and untrained people with PD has been determined, finding that verbal secondary tasks have a greater impact on motor performance than visual or motor secondary tasks with the arms (San Martín Valenzuela et al., 2020a,b). In this way, the balanceHOME protocol pretends to resolve methodological issues and research questions relevant to the advancement of physical rehabilitation of balance in the population with Parkinson’s disease.

This protocol will make it possible to facilitate highly challenging functional balance exercises for people with PD at their homes, using common materials of daily use. Similarly, the objective assessment methodology and the balanceHOME protocol will allow us to identify small changes in the balance performance of people with different stages of disease severity and to determine the effectiveness of the home program in people without and with cognitive impairment, which has not been done until now as we will be able to establish the scope that physiotherapy has when it is applied in patients with motor and cognitive failures. Unlike the authors who report improvements below 50% in objective parameters, we think that the structured and progressive dynamics of balanceHOME program can exceed these values of improvement in both dynamic and static balance in single and dual conditions, while performing secondary cognitive and motor tasks of the arms during standing balance maintenance. Furthermore, due to the convenience of home rehabilitation, we think that the effects may achieve better retention during the follow-up period than standard control therapy. This protocol aims to promote change in functional balance physiotherapy both in evaluation and rehabilitation, bringing day-to-day conditions closer to clinical practice.

## 4. Ethics and dissemination

The Human Research Ethics Committee of the Experimental Research Ethics Commission of the University of Valencia approved all the procedures that will perform (Procedure N° 1686831) in accordance with the principles of the World Medical Association’s Declaration of Helsinki and the Council of Europe Convention regarding human rights. The physiotherapists in charge of evaluating the eligibility criteria will be responsible for obtaining written informed consent from the participants before the first tests started, which included a detailed explanation of the research milestones, the personal data protection procedures, and the images agreement if necessary to support the dissemination of study results. The signed documents will be filed in a locked cabinet in the office of the principal researcher at the University of Valencia. All the participants’ personal data will be kept completely anonymous in the scientific publications of this study. All results from the trial will be published in international peer-reviewed scientific journals, regardless these may result being negative or inconclusive.

## Ethics statement

The studies involving human participants were reviewed and approved by Human Research Ethics Committee of the Experimental Research Ethics Commission of the University of Valencia. The patients/participants provided their written informed consent to participate in this study.

## Author contributions

SMG, CSV, and JMT: conceptualization, visualization, and writing - original draft. CSV and JMT: project administration and supervision. SMG, CSV, JMT, VAA, CDA, and MVN: writing – review, and editing. All authors contributed to the article and approved the submitted version.

## Funding

The authors CSV and JMT are part of the project with number PID2021-124.4180B-100 funded by MCIN/AEI/10.13.039/501.100.011.033 and by “ERDF A way of making Europe.”

## Conflict of interest

The authors declare that the research was conducted in the absence of any commercial or financial relationships that could be construed as a potential conflict of interest.

## Publisher’s note

All claims expressed in this article are solely those of the authors and do not necessarily represent those of their affiliated organizations, or those of the publisher, the editors and the reviewers. Any product that may be evaluated in this article, or claim that may be made by its manufacturer, is not guaranteed or endorsed by the publisher.
