# Supplementary material for: Functional balance training in people with Parkinson’s disease: a protocol of balanceHOME randomized control trial with crossover
Source: Front Aging Neurosci. 2023 Apr 26;15:1137360. doi: 10.3389/fnagi.2023.1137360 (PMC10231658; doi:10.3389/fnagi.2023.1137360)
Supplement: Supplementary file 1 [file Data_Sheet_1.pdf]

STANDARD PROTOCOL ITEMS: RECOMMENDATIONS FOR INTERVENTIONAL TRIALS

Functional Balance training in people with Parkinson's disease: protocol of balanceHOME randomized control trial with crossover

Section/item	Item No	Description	Page Number on which item is reported in the original draft
**Administrative information**			
Title	1	Descriptive title identifying the study design, population, interventions, and, if applicable, trial acronym	Page 1
Trial registration	2a	Trial identifier and registry name. If not yet registered, name of intended registry<br>ClinicalTrials.gov Identifier: NCT04963894	Page 1, line 19.<br>Page 3, line 99
	2b	All items from the World Health Organization Trial Registration Data Set	N/A
Protocol version	3	Date and version identifier<br>Date of registration: July 15, 2021<br>Date last update: December 8, 2022	
Funding	4	Sources and types of financial, material, and other support<br>This study has not received funding.<br>The resources and materials used in this study belong to the affiliation institutions of the authors of this manuscript.	Page 8, line 318
Roles and responsibilities	5a	Names, affiliations, and roles of protocol contributors	Page 1, lines 1-10.<br>Page 8, lines 340-342.
	5b	Name and contact information for the trial sponsor<br>Principal researcher: Constanza San Martín, PhD.<br>constanza.martin@uv.es	N/A

	5c	Role of study sponsor and funders, if any, in study design; collection, management, analysis, and interpretation of data; writing of the report; and the decision to submit the report for publication, including whether they will have ultimate authority over any of these activities: None.	N/A
	5d	Composition, roles, and responsibilities of the coordinating centre, steering committee, endpoint adjudication committee, data management team, and other individuals or groups overseeing the trial, if applicable (see Item 21a for data monitoring committee).	N/A
**Introduction**			
Background and rationale	6a	Description of research question and justification for undertaking the trial, including summary of relevant studies (published and unpublished) examining benefits and harms for each intervention	Page 2-3. Lines 38-83
	6b	Explanation for choice of comparators	Page 3. Lines 102-110.
Objectives	7	Specific objectives or hypotheses	Page 2-3. Lines 83-91.
Trial design	8	Description of trial design including type of trial (eg, parallel group, crossover, factorial, single group), allocation ratio, and framework (eg, superiority, equivalence, noninferiority, exploratory)	Page 3. Lines 94-98.
**Methods: Participants, interventions, and outcomes**			
Study setting	9	Description of study settings (eg, community clinic, academic hospital) and list of countries where data will be collected. Reference to where list of study sites can be obtained	Page 3. Lines 112-117.
Eligibility criteria	10	Inclusion and exclusion criteria for participants. If applicable, eligibility criteria for study centres and individuals who will perform the interventions (eg, surgeons, psychotherapists)	Page 3. Lines 118-126
Interventions	11a	Interventions for each group with sufficient detail to allow replication, including how and when they will be administered	Page 4. Lines 127-156. Table 1.

	11b	Criteria for discontinuing or modifying allocated interventions for a given trial participant (eg, drug dose change in response to harms, participant request, or improving/worsening disease)	Page 7. Line 264-272.
	11c	Strategies to improve adherence to intervention protocols, and any procedures for monitoring adherence (eg, drug tablet return, laboratory tests)	Page 7. Lines 264-272.
	11d	Relevant concomitant care and interventions that are permitted or prohibited during the trial	Page 3. Lines 123-124.<br>Page 7. Lines 268-270.
Outcomes	12	Primary, secondary, and other outcomes, including the specific measurement variable (eg, systolic blood pressure), analysis metric (eg, change from baseline, final value, time to event), method of aggregation (eg, median, proportion), and time point for each outcome. Explanation of the clinical relevance of chosen efficacy and harm outcomes is strongly recommended	Page 4-5. Lines 157-202 Table 2.
Participant timeline	13	Time schedule of enrolment, interventions (including any run-ins and washouts), assessments, and visits for participants. A schematic diagram is highly recommended (see Figure)	Page 3. Lines 102-110. Figure 1.
Sample size	14	Estimated number of participants needed to achieve study objectives and how it was determined, including clinical and statistical assumptions supporting any sample size calculations	Page 5. Lines 215-222.
Recruitment	15	Strategies for achieving adequate participant enrolment to reach target sample size	Page 5-6. Lines 222-230.
**Methods: Assignment of interventions (for controlled trials)**			
Allocation:			
Sequence generation	16a	Method of generating the allocation sequence (eg, computer-generated random numbers), and list of any factors for stratification. To reduce predictability of a random sequence, details of any planned restriction (eg, blocking) should be provided in a separate document that is unavailable to those who enrol participants or assign interventions	Page 6. Lines 231-235.

Allocation concealment mechanism	16b	Mechanism of implementing the allocation sequence (eg, central telephone; sequentially numbered, opaque, sealed envelopes), describing any steps to conceal the sequence until interventions are assigned	Page 6. Lines 231-235.
Implementation	16c	Who will generate the allocation sequence, who will enrol participants, and who will assign participants to interventions	Page 6. Lines 231-235.
Blinding (masking)	17a	Who will be blinded after assignment to interventions (eg, trial participants, care providers, outcome assessors, data analysts), and how	Page 6. Lines 235-240.
	17b	If blinded, circumstances under which unblinding is permissible, and procedure for revealing a participant's allocated intervention during the trial	N/A.
**Methods: Data collection, management, and analysis**			
Data collection methods	18a	Plans for assessment and collection of outcome, baseline, and other trial data, including any related processes to promote data quality (eg, duplicate measurements, training of assessors) and a description of study instruments (eg, questionnaires, laboratory tests) along with their reliability and validity, if known. Reference to where data collection forms can be found, if not in the protocol	Page 6. Lines 241-250.
	18b	Plans to promote participant retention and complete follow-up, including list of any outcome data to be collected for participants who discontinue or deviate from intervention protocols.	Page 7. Lines 263-272.
Data management	19	Plans for data entry, coding, security, and storage, including any related processes to promote data quality (eg, double data entry; range checks for data values). Reference to where details of data management procedures can be found, if not in the protocol	Page 6. Lines 241-250. Page 8. Lines 324-335.
Statistical methods	20a	Statistical methods for analysing primary and secondary outcomes. Reference to where other details of the statistical analysis plan can be found, if not in the protocol	Page 6. Lines 251-262.
	20b	Methods for any additional analyses (eg, subgroup and adjusted analyses)	N/A.

	20c	Definition of analysis population relating to protocol non-adherence (eg, as randomised analysis), and any statistical methods to handle missing data (eg, multiple imputation)	Page 6. Lines 251-262.
**Methods: Monitoring**			
Data monitoring	21a	Composition of data monitoring committee (DMC); summary of its role and reporting structure; statement of whether it is independent from the sponsor and competing interests; and reference to where further details about its charter can be found, if not in the protocol. Alternatively, an explanation of why a DMC is not needed	Page 7. Lines 263-272.
	21b	Description of any interim analyses and stopping guidelines, including who will have access to these interim results and make the final decision to terminate the trial	N/A
Harms	22	Plans for collecting, assessing, reporting, and managing solicited and spontaneously reported adverse events and other unintended effects of trial interventions or trial conduct	Page 6. Lines 263-272.
Auditing	23	Frequency and procedures for auditing trial conduct, if any, and whether the process will be independent from investigators and the sponsor	N/A
**Ethics and dissemination**			
Research ethics approval	24	Plans for seeking research ethics committee/institutional review board (REC/IRB) approval. The Human Research Ethics Committee of the Experimental Research Ethics Commission of the University of Valencia approved all the procedures that will perform (Procedure Nº 1686831).	Page 8. Lines 324-335. Supplementary material 2.
Protocol amendments	25	Plans for communicating important protocol modifications (eg, changes to eligibility criteria, outcomes, analyses) to relevant parties (eg, investigators, REC/IRBs, trial participants, trial registries, journals, regulators)	N/A
Consent or assent	26a	Who will obtain informed consent or assent from potential trial participants or authorised surrogates, and how (see Item 32)	Page 8. Lines 328-333.

	26b	Additional consent provisions for collection and use of participant data and biological specimens in ancillary studies, if applicable	Page 8. Lines 328-333.
Confidentiality	27	How personal information about potential and enrolled participants will be collected, shared, and maintained in order to protect confidentiality before, during, and after the trial	Page 8. Lines 333-335.
Declaration of interests	28	Financial and other competing interests for principal investigators for the overall trial and each study site	Page 8. Lines 343-344.
Access to data	29	Statement of who will have access to the final trial dataset, and disclosure of contractual agreements that limit such access for investigators	Page 8. Lines 333-335.
Ancillary and post-trial care	30	Provisions, if any, for ancillary and post-trial care, and for compensation to those who suffer harm from trial participation	N/A
Dissemination policy	31a	Plans for investigators and sponsor to communicate trial results to participants, healthcare professionals, the public, and other relevant groups (eg, via publication, reporting in results databases, or other data sharing arrangements), including any publication restrictions	Page 7. Lines 271-272.<br>Page 8. Lines 324-335.
	31b	Authorship eligibility guidelines and any intended use of professional writers.<br>The contribution of each author specifies the page and indicated lines. No help from outside writing services or professional writers was used.	Page 8. Lines 339-342.
	31c	Plans, if any, for granting public access to the full protocol, participant-level dataset, and statistical code	Page 7. Lines 271-272.<br>Page 8. Lines 324-335.
**Appendices**			
Informed consent materials	32	Model consent form and other related documentation given to participants and authorised surrogates	Supplementary material 3
Biological specimens	33	Plans for collection, laboratory evaluation, and storage of biological specimens for genetic or molecular analysis in the current trial and for future use in ancillary studies, if applicable	N/A

*It is strongly recommended that this checklist be read in conjunction with the SPIRIT 2013 Explanation & Elaboration for important clarification on the items. Amendments to the

protocol should be tracked and dated. The SPIRIT checklist is copyrighted by the SPIRIT Group under the Creative Commons "Attribution-NonCommercial-NoDerivs 3.0 Unported" license.
