# Supplementary material for: Functional balance training in people with Parkinson’s disease: a protocol of balanceHOME randomized control trial with crossover
Source: Front Aging Neurosci. 2023 Apr 26;15:1137360. doi: 10.3389/fnagi.2023.1137360 (PMC10231658; doi:10.3389/fnagi.2023.1137360)
Supplement: Supplementary file 2 [file Data_Sheet_2.pdf]

El comité Ético de Investigación en Humanos de la Comisión de Ética en Investigación Experimental de la Universitat de València,

CERTIFICA:

Que el Comité d'Ètica d'Investigació en Humans , en la reunión celebrada el día , una vez estudiado el proyecto de tesis doctoral : *"EFECTOS DE LA REHABILITACIÓN DOMICILIARIA DEL EQUILIBRIO BASADO EN EJERCICIOS FUNCIONALES EN PERSONAS CON ENFERMEDAD DE PARKINSON: ENSAYO CLÍNICO ALEATORIZADO "*, con número de registro1686831 .

Cuyo/a responsable es D/Dña.

CONSTANZA ISABEL SAN MARTIN VALENZUELA , dirigida por D/Dña. CONSTANZA ISABEL SAN MARTIN VALENZUELA

ha acordado informar favorablemente el mismo.

Y para que conste, se firma el presente certificado

Av. Blasco Ibáñez, 13 tel: 963864109 vicerec.investigacio@uv.es
València 46010 fax: 963983221 www.uv.es/serinves

Firmado digitalmente por
PEDRO JESUS PEREZ ZAFRILLA
Cargo: Presidente del Comité de Ética de la Investigación en Humanos
Fecha: 03/07/2021 23:29:32 CEST

The Ethics Committee of Research in Humans of the Ethics Commission in Experimental Research of University of Valencia,

CERTIFY:

Hereby certify that the Ethics Committee of Research in Humans, in the session which took place on , analysed the project of doctoral thesis entitled *"EFFECTS OF HOME REHABILITATION OF BALANCE BASED ON FUNCTIONAL EXERCISES IN PEOPLE WITH PARKINSON'S DISEASE: RANDOMIZED CLINICAL TRIAL "*, with register code 1686831 .
Whose researcher in charge is CONSTANZA ISABEL SAN MARTIN VALENZUELA , and agreed with this project.

And in witness whereof, I hereby sign this certificate

Av. Blasco Ibáñez, 13 tel: 963864109 vicerec.investigacio@uv.es
València 46010 fax: 963983221 www.uv.es/serinves

Comité d'Ètica d'Investigació en Humans de la Comissió d'Ètica en Investigació Experimental de la Universitat de València,

CERTIFICA:

Que Comité d'Ètica d'Investigació en Humans , en la reunió que tingué lloc el dia , una vegada estudiat el projecte de tesi doctoral titulat:*"EFECTES DE LA REHABILITACIÓ DOMICILIÀRIA DE L'EQUILIBRI BASAT EN EXERCICIS FUNCIONALS EN PERSONES AMB MALALTIA DE PARKINSON: ASSAIG CLÍNIC ALEATORITZAT "*, amb codi de registre 1686831 .

que té com a responsable

CONSTANZA ISABEL SAN MARTIN VALENZUELA , i que va dirigir CONSTANZA ISABEL SAN MARTIN VALENZUELA , ha acordat emetre'n un informe favorablement .

I perquè així conste, signa aquest certificat.

Av. Blasco Ibáñez, 13 tel: 963864109 vicerec.investigacio@uv.es
València 46010 fax: 963983221 www.uv.es/serinves
