# Supplementary material for: Functional balance training in people with Parkinson’s disease: a protocol of balanceHOME randomized control trial with crossover
Source: Front Aging Neurosci. 2023 Apr 26;15:1137360. doi: 10.3389/fnagi.2023.1137360 (PMC10231658; doi:10.3389/fnagi.2023.1137360)
Supplement: Supplementary file 3 [file Data_Sheet_3.pdf]

DOCUMENTO DE CONSENTIMIENTO INFORMADO
Y COMPROMISO DE CONFIDENCIALIDAD

1.- INFORMACIÓN AL SUJETO DE EXPERIMENTACIÓN.

El proyecto de investigación para el cual le pedimos su participación se titula:
"EFECTOS DE LA REHABILITACIÓN DOMICILIARIA DEL EQUILIBRIO BASADO EN EJERCICIOS FUNCIONALES EN PERSONAS CON ENFERMEDAD DE PARKINSON: ENSAYO CLÍNICO ALEATORIZADO".

Para que usted pueda participar en este estudio es necesario contar con su consentimiento, y que conozca la información básica necesaria para que dicho consentimiento pueda considerarse verdaderamente informado. Por ello, le ruego que lea detenidamente la siguiente información. Si tuviera alguna duda exprésela, antes de firmar este documento, al investigador principal del proyecto, bien personalmente, bien a través del teléfono o por correo electrónico. Los datos del investigador principal del proyecto aparecen también en el presente documento.

La información básica que debe conocer es la siguiente:

a) *Objetivo del estudio*: Determinar si los efectos de la rehabilitación domiciliaria del equilibrio funcional sobre la biomecánica del equilibrio y la marcha, la calidad de vida, el estado cognitivo, y el desempeño físico general, son mayores que los efectos observados en un programa de fisioterapia convencional grupal y presencial y que los efectos de un programa de fisioterapia convencional domiciliar desarrollado de manera telemática.

b) *Metodología a utilizar para el estudio, tipo de colaboración que se espera de usted y duración de dicha colaboración*: Se le solicita autorización para participar en un programa de fisioterapia para la enfermedad de Parkinson que pretende mejorar su equilibrio. Dicho programa se realizará en su propio domicilio. Tendrá que realizar un total de 2 sesiones semanales de 60 minutos, en donde se practicarán diferentes ejercicios para mejorar el equilibrio durante actividades de la vida diaria. Con el fin de determinar el efecto de la terapia, se requiere que participe en tres evaluaciones de 90 minutos. Las evaluaciones se llevarán a cabo en la Universidad de Valencia antes de que comience la intervención, al terminarla y a las 8 semanas. En ellas usted debe contestar algunos cuestionarios acerca de su estado de salud y realizar pruebas de equilibrio y marcha de uso frecuente a nivel clínico.

c) *Procedimientos preventivos, diagnósticos y/o terapéuticos disponibles alternativos a los que se investigan con este estudio*: NO PROCEDE.

d) *Posibles molestias y riesgos de su participación en el estudio*: NO PROCEDE. Este estudio no supone ningún riesgo para su salud.

e) *Medidas para responder a los acontecimientos adversos*: NO PROCEDE.

f) *Medidas para asegurar una compensación adecuada en el caso de que usted sufra algún daño*: NO PROCEDE.

g) *Beneficios que se espera obtener con la investigación*: Las técnicas fisioterápicas empleadas podrían mejorar su función motora, equilibrio, marcha y calidad de vida. Sin embargo, existe la probabilidad que no experimente ningún cambio. Además, este estudio pretende mejorar el tratamiento fisioterápico de futuras personas con Enfermedad de Parkinson.

h) *Consecuencias de la no participación*: La participación en este estudio es voluntaria y puede cancelarse en cualquier momento. Si rechaza participar, no habrá consecuencias negativas para usted. En caso de que decida no participar en el mismo, esto no modificará el

trato y seguimiento que usted realiza con el equipo investigador, su médico, su fisioterapeuta, o con el resto del personal sanitario.

i) *Posibilidad de retirada en cualquier momento y consecuencias*: Usted puede retirarse del proyecto en cualquier momento firmando la revocación del consentimiento que se incluye al final del documento. Su retirada no tendrá ninguna consecuencia negativa para usted, y será aceptada sin problemas por el equipo investigador. Si se retira del proyecto, puede decidir si los datos utilizados hasta ese momento deben borrarse o si se pueden seguir utilizando tras haberlos convertido en anónimos.

j) *¿Quién ha financiado el estudio?*: Sin financiación externa.

k) *¿Qué institución lo realiza?*: Universitat de València y Asociación Parkinson Valencia.

l) *Gratuidad por la participación*: Los participantes de la intervención no recibirán ningún tipo de compensación económica.

m) *Previsión de uso posterior de los resultados*: Los resultados del estudio solamente se utilizarán con fines de docencia, investigación y/o publicaciones científicas, se respetará siempre la debida anonimización de los datos de carácter personal, de modo que los participantes de la investigación no resultarán identificados o identificables.

n) *Equipo investigador*: Constanza San Martín, José M. Tomás, Sara Monleón, Vivina Aranda, Concepción de Salazar Antón y Manuel Villanueva.

o) *Datos de contacto del investigador principal para aclaraciones o consultas*: Podrá solicitar cualquier explicación que desee sobre cualquier aspecto del estudio contactando en el teléfono 633105601 con la profesora/investigadora Constanza San Martín, por correo electrónico constanza.martin@uv.es o acudiendo a su despacho en la Facultat de Fisioteràpia de Valencia (C/Gascó Oliag nº5). También puede dirigir sus consultas a Sara Monleón al teléfono 680807815 o mediante el correo electrónico samongui@alumni.uv.es

p) El proyecto se realizará siguiendo los criterios éticos internacionales recogidos en la Declaración de Helsinki.

2.- COMPROMISO DE CONFIDENCIALIDAD.

a) *Medidas para asegurar el respeto a la vida privada y a la confidencialidad de los datos personales*: Se han adoptado las medidas oportunas para garantizar la completa confidencialidad de los datos personales de los sujetos de experimentación que participen en este estudio, de acuerdo con la Ley De Protección de Datos de Carácter Personal (LOPD) 3/2018, de 5 de diciembre. Todos sus datos e información médica que proporcione serán tratados con absoluta confidencialidad por los investigadores. Si los resultados del estudio fueran susceptibles de publicación en revistas científicas, en ningún momento se proporcionarán datos personales de las personas que han colaborado en esta investigación.

b) *Medidas para acceder a la información relevante para usted que surjan de la investigación o de los resultados totales*: Sepa que tiene derecho a acceder a la información generada sobre usted en el estudio, solicitándola al investigador principal.

c) *Medidas tomadas por tratarse de un estudio anonimizado*: Se ha establecido un sistema de anonimización efectivo que no permite la identificación posterior del participante. Los consentimiento otorgados y los cuestionarios utilizados en el estudio se custodiarán de manera separada. El uso de los resultados será con fines de docencia, investigación y/o publicación, y se respetará la anonimización de los datos personales.

3.- CONSENTIMIENTO **(EJEMPLAR PARA EL/LA PARTICIPANTE)**

En el caso de que el sujeto de experimentación sea mayor de edad:

Don/Doña ____,

mayor de edad, titular del DNI : ____________________, por el presente documento

manifiesto que:

En el caso de que el sujeto de experimentación sea menor de edad o incapaz de obrar:

Don/Doña ____,

mayor de edad, titular del DNI : ________________________,

☐ padre, ☐ madre, ☐ tutor legal

de ____,

por el presente documento manifiesto que:

He sido informado/a de las características del Proyecto de Investigación titulado: “EFECTOS DE LA REHABILITACIÓN DOMICILIARIA DEL EQUILIBRIO BASADO EN EJERCICIOS FUNCIONALES EN PERSONAS CON ENFERMEDAD DE PARKINSON: ENSAYO CLÍNICO ALEATORIZADO”.
He leído tanto el apartado 1 del presente documento titulado “información al sujeto de experimentación”, como el apartado 2 titulado “compromiso de confidencialidad”, y he podido formular las dudas que me han surgido al respecto. Considero que he entendido dicha información.
Estoy informado/a de la posibilidad de retirarme en cualquier momento del estudio.

En virtud de tales condiciones, consiento participar en este estudio.

Y en prueba de conformidad, firmo el presente documento en el lugar y fecha que se indican a continuación.

Valencia, ________ de ______________ de 20___.

Nombre y apellidos del / de la participante:	*Nombre y apellidos del padre, madre o tutor (en el caso de menores o incapaces)*:	*Nombre y apellidos del investigador principal*:
Firma:	Firma:	Firma:

Si el sujeto del estudio es un adolescente capaz intelectual y emocionalmente de entre 12 y 16 años debe de ser oída su opinión y autorizar su participación en el estudio firmando también este consentimiento. Cuando se trate de menores no incapaces ni incapacitados, pero emancipados o con 16 años cumplidos, no cabe prestar el consentimiento por representación y será el propio sujeto del estudio quien firmará el consentimiento (Ley 41/2002).

REVOCACIÓN DEL CONSENTIMIENTO

Revoco el consentimiento prestado en fecha ________________________ para participar en el proyecto titulado "EFECTOS DE LA REHABILITACIÓN DOMICILIARIA DEL EQUILIBRIO BASADO EN EJERCICIOS FUNCIONALES EN PERSONAS CON ENFERMEDAD DE PARKINSON: ENSAYO CLÍNICO ALEATORIZADO" y, para que así conste, firmo la presente revocación.

En Valencia, a _______ de ______________________ de 20___.

Nombre y apellidos del / de la participante:<br><br><br>Firma:	*Nombre y apellidos del padre, madre o tutor (en el caso de menores o incapaces)*:<br><br><br>Firma:	*Nombre y apellidos del investigador principal*:<br><br><br>Firma:

3.- CONSENTIMIENTO **(EJEMPLAR PARA EL EQUIPO INVESTIGADOR)**

En el caso de que el sujeto de experimentación sea mayor de edad:

Don/Doña ____,

mayor de edad, titular del DNI : ____________________, por el presente documento manifiesto que:

En el caso de que el sujeto de experimentación sea menor de edad o incapaz de obrar:

Don/Doña ____,

mayor de edad, titular del DNI : ___________________________,

☐ padre, ☐ madre, ☐ tutor legal

de ____,

por el presente documento manifiesto que:

He sido informado/a de las características del Proyecto de Investigación titulado: "EFECTOS DE LA REHABILITACIÓN DOMICILIARIA DEL EQUILIBRIO BASADO EN EJERCICIOS FUNCIONALES EN PERSONAS CON ENFERMEDAD DE PARKINSON: ENSAYO CLÍNICO ALEATORIZADO".
He leído tanto el apartado 1 del presente documento titulado "información al sujeto de experimentación", como el apartado 2 titulado "compromiso de confidencialidad", y he podido formular las dudas que me han surgido al respecto. Considero que he entendido dicha información.
Estoy informado/a de la posibilidad de retirarme en cualquier momento del estudio.

En virtud de tales condiciones, consiento participar en este estudio.

Y en prueba de conformidad, firmo el presente documento en el lugar y fecha que se indican a continuación.

Valencia, ________ de ______________ de 20__.

Nombre y apellidos del / de la participante:	*Nombre y apellidos del padre, madre o tutor (en el caso de menores o incapaces)*:	*Nombre y apellidos del investigador principal*:
Firma:	Firma:	Firma:

Si el sujeto del estudio es un adolescente capaz intelectual y emocionalmente de entre 12 y 16 años debe de ser oída su opinión y autorizar su participación en el estudio firmando también este consentimiento. Cuando se trate de menores no incapaces ni incapacitados, pero emancipados o con 16 años cumplidos, no cabe prestar el consentimiento por representación y será el propio sujeto del estudio quien firmará el consentimiento (Ley 41/2002).

REVOCACIÓN DEL CONSENTIMIENTO

Revoco el consentimiento prestado en fecha ________________________ para participar en el proyecto titulado "EFECTOS DE LA REHABILITACIÓN DOMICILIARIA DEL EQUILIBRIO BASADO EN EJERCICIOS FUNCIONALES EN PERSONAS CON ENFERMEDAD DE PARKINSON: ENSAYO CLÍNICO ALEATORIZADO" y, para que así conste, firmo la presente revocación.

En Valencia, a _______ de ______________________ de 20__.

Nombre y apellidos del / de la participante:<br><br>Firma:	*Nombre y apellidos del padre, madre o tutor (en el caso de menores o incapaces)*:<br><br>Firma:	*Nombre y apellidos del investigador principal*:<br><br>Firma:

DOCUMENTO DE CONSENTIMIENTO INFORMADO
Y COMPROMISO DE CONFIDENCIALIDAD

1.- INFORMACIÓN AL SUJETO DE EXPERIMENTACIÓN.

El proyecto de investigación para el cual le pedimos su participación se titula:
"EFECTOS DE LA REHABILITACIÓN DOMICILIARIA DEL EQUILIBRIO BASADO EN EJERCICIOS FUNCIONALES EN PERSONAS CON ENFERMEDAD DE PARKINSON: ENSAYO CLÍNICO ALEATORIZADO".

Para que usted pueda participar en este estudio es necesario contar con su consentimiento, y que conozca la información básica necesaria para que dicho consentimiento pueda considerarse verdaderamente informado. Por ello, le ruego que lea detenidamente la siguiente información. Si tuviera alguna duda exprésela, antes de firmar este documento, al investigador principal del proyecto, bien personalmente, bien a través del teléfono o por correo electrónico. Los datos del investigador principal del proyecto aparecen también en el presente documento.

La información básica que debe conocer es la siguiente:

a) *Objetivo del estudio*: Determinar si los efectos de la rehabilitación domiciliaria del equilibrio funcional sobre la biomecánica del equilibrio y la marcha, la calidad de vida, el estado cognitivo, y el desempeño físico general, son mayores que los efectos observados en un programa de fisioterapia convencional grupal y presencial y que los efectos de un programa de fisioterapia convencional domiciliar desarrollado de manera telemática.

b) *Metodología a utilizar para el estudio, tipo de colaboración que se espera de usted y duración de dicha colaboración*: Se le solicita autorización para evaluar los efectos de la fisioterapia que realiza en Asociación Parkinson Valencia en su grupo de terapia habitual. Tendrá que completar un total de 2 sesiones semanales de 60 minutos, en donde se practicarán los ejercicios habituales de la fisioterapia. Con el fin de determinar el efecto de la terapia, se requiere que participe en tres evaluaciones de 90 minutos. Las evaluaciones se llevarán a cabo en la Universidad de Valencia antes de que comience la intervención, al terminarla y a las 8 semanas. En ellas usted debe contestar algunos cuestionarios acerca de su estado de salud y realizar pruebas de equilibrio y marcha de uso frecuente a nivel clínico.

c) *Procedimientos preventivos, diagnósticos y/o terapéuticos disponibles alternativos a los que se investigan con este estudio*: NO PROCEDE.

d) *Posibles molestias y riesgos de su participación en el estudio*: NO PROCEDE. Este estudio no supone ningún riesgo para su salud.

e) *Medidas para responder a los acontecimientos adversos*: NO PROCEDE.

f) *Medidas para asegurar una compensación adecuada en el caso de que usted sufra algún daño*: NO PROCEDE.

g) *Beneficios que se espera obtener con la investigación*: Las técnicas fisioterápicas empleadas podrían mejorar su función motora, equilibrio, marcha y calidad de vida. Sin embargo, existe la probabilidad que no experimente ningún cambio. Además, este estudio pretende mejorar el tratamiento fisioterápico de futuras personas con Enfermedad de Parkinson.

h) *Consecuencias de la no participación*: La participación en este estudio es voluntaria y puede cancelarse en cualquier momento. Si rechaza participar, no habrá consecuencias negativas para usted. En caso de que decida no participar en el mismo, esto no modificará el

trato y seguimiento que usted realiza con el equipo investigador, su médico, su fisioterapeuta, o con el resto del personal sanitario.

i) *Posibilidad de retirada en cualquier momento y consecuencias*: Usted puede retirarse del proyecto en cualquier momento firmando la revocación del consentimiento que se incluye al final del documento. Su retirada no tendrá ninguna consecuencia negativa para usted, y será aceptada sin problemas por el equipo investigador. Si se retira del proyecto, puede decidir si los datos utilizados hasta ese momento deben borrarse o si se pueden seguir utilizando tras haberlos convertido en anónimos.

j) *¿Quién ha financiado el estudio?*: Sin financiación externa.

k) *¿Qué institución lo realiza?*: Universitat de València y Asociación Parkinson Valencia.

l) *Gratuidad por la participación*: Los participantes de la intervención no recibirán ningún tipo de compensación económica.

m) *Previsión de uso posterior de los resultados*: Los resultados del estudio solamente se utilizarán con fines de docencia, investigación y/o publicaciones científicas, se respetará siempre la debida anonimización de los datos de carácter personal, de modo que los participantes de la investigación no resultarán identificados o identificables.

n) *Equipo investigador*: Constanza San Martín, José M. Tomás, Sara Monleón, Vivina Aranda, Concepción de Salazar Antón y Manuel Villanueva.

o) *Datos de contacto del investigador principal para aclaraciones o consultas*: Podrá solicitar cualquier explicación que desee sobre cualquier aspecto del estudio contactando en el teléfono 633105601 con la profesora/investigadora Constanza San Martín, por correo electrónico constanza.martin@uv.es o acudiendo a su despacho en la Facultat de Fisioteràpia de Valencia (C/Gascó Oliag nº5). También puede dirigir sus consultas a Sara Monleón al teléfono 680807815 o mediante el correo electrónico samongui@alumni.uv.es

p) El proyecto se realizará siguiendo los criterios éticos internacionales recogidos en la Declaración de Helsinki.

2.- COMPROMISO DE CONFIDENCIALIDAD.

a) *Medidas para asegurar el respeto a la vida privada y a la confidencialidad de los datos personales*: Se han adoptado las medidas oportunas para garantizar la completa confidencialidad de los datos personales de los sujetos de experimentación que participen en este estudio, de acuerdo con la Ley De Protección de Datos de Carácter Personal (LOPD) 3/2018, de 5 de diciembre. Todos sus datos e información médica que proporcione serán tratados con absoluta confidencialidad por los investigadores. Si los resultados del estudio fueran susceptibles de publicación en revistas científicas, en ningún momento se proporcionarán datos personales de las personas que han colaborado en esta investigación.

b) *Medidas para acceder a la información relevante para usted que surjan de la investigación o de los resultados totales*: Sepa que tiene derecho a acceder a la información generada sobre usted en el estudio, solicitándola al investigador principal.

c) *Medidas tomadas por tratarse de un estudio anonimizado*: Se ha establecido un sistema de anonimización efectivo que no permite la identificación posterior del participante. Los consentimiento otorgados y los cuestionarios utilizados en el estudio se custodiarán de manera separada. El uso de los resultados será con fines de docencia, investigación y/o publicación, y se respetará la anonimización de los datos personales.

3.- CONSENTIMIENTO **(EJEMPLAR PARA EL/LA PARTICIPANTE)**

En el caso de que el sujeto de experimentación sea mayor de edad:

Don/Doña ____,

mayor de edad, titular del DNI : ____________________, por el presente documento

manifiesto que:

En el caso de que el sujeto de experimentación sea menor de edad o incapaz de obrar:

Don/Doña ____,

mayor de edad, titular del DNI : _________________________,

☐ padre, ☐ madre, ☐ tutor legal

de ____,

por el presente documento manifiesto que:

He sido informado/a de las características del Proyecto de Investigación titulado: "EFECTOS DE LA REHABILITACIÓN DOMICILIARIA DEL EQUILIBRIO BASADO EN EJERCICIOS FUNCIONALES EN PERSONAS CON ENFERMEDAD DE PARKINSON: ENSAYO CLÍNICO ALEATORIZADO".
He leído tanto el apartado 1 del presente documento titulado "información al sujeto de experimentación", como el apartado 2 titulado "compromiso de confidencialidad", y he podido formular las dudas que me han surgido al respecto. Considero que he entendido dicha información.
Estoy informado/a de la posibilidad de retirarme en cualquier momento del estudio.

En virtud de tales condiciones, consiento participar en este estudio.

Y en prueba de conformidad, firmo el presente documento en el lugar y fecha que se indican a continuación.

Valencia, ________ de _______________ de 20___.

Nombre y apellidos del / de la participante:<br><br><br>Firma:	*Nombre y apellidos del padre, madre o tutor (en el caso de menores o incapaces)*:<br><br><br>Firma:	*Nombre y apellidos del investigador principal*:<br><br><br>Firma:

Si el sujeto del estudio es un adolescente capaz intelectual y emocionalmente de entre 12 y 16 años debe de ser oída su opinión y autorizar su participación en el estudio firmando también este consentimiento. Cuando se trate de menores no incapaces ni incapacitados, pero emancipados o con 16 años cumplidos, no cabe prestar el consentimiento por representación y será el propio sujeto del estudio quien firmará el consentimiento (Ley 41/2002).

REVOCACIÓN DEL CONSENTIMIENTO

Revoco el consentimiento prestado en fecha ________________________ para participar en el proyecto titulado “EFECTOS DE LA REHABILITACIÓN DOMICILIARIA DEL EQUILIBRIO BASADO EN EJERCICIOS FUNCIONALES EN PERSONAS CON ENFERMEDAD DE PARKINSON: ENSAYO CLÍNICO ALEATORIZADO” y, para que así conste, firmo la presente revocación.

En Valencia, a _______ de ______________________ de 20___.

Nombre y apellidos del / de la participante:<br><br><br><br><br>Firma:	*Nombre y apellidos del padre, madre o tutor (en el caso de menores o incapaces)*:<br><br><br>Firma:	*Nombre y apellidos del investigador principal*:<br><br><br><br><br>Firma:

3.- CONSENTIMIENTO **(EJEMPLAR PARA EL EQUIPO INVESTIGADOR)**

En el caso de que el sujeto de experimentación sea mayor de edad:

Don/Doña ____,

mayor de edad, titular del DNI : ______________, por el presente documento manifiesto que:

En el caso de que el sujeto de experimentación sea menor de edad o incapaz de obrar:

Don/Doña ____,

mayor de edad, titular del DNI : __________________,

☐ padre, ☐ madre, ☐ tutor legal

de ____,

por el presente documento manifiesto que:

He sido informado/a de las características del Proyecto de Investigación titulado: "EFECTOS DE LA REHABILITACIÓN DOMICILIARIA DEL EQUILIBRIO BASADO EN EJERCICIOS FUNCIONALES EN PERSONAS CON ENFERMEDAD DE PARKINSON: ENSAYO CLÍNICO ALEATORIZADO".
He leído tanto el apartado 1 del presente documento titulado "información al sujeto de experimentación", como el apartado 2 titulado "compromiso de confidencialidad", y he podido formular las dudas que me han surgido al respecto. Considero que he entendido dicha información.
Estoy informado/a de la posibilidad de retirarme en cualquier momento del estudio.

En virtud de tales condiciones, consiento participar en este estudio.

Y en prueba de conformidad, firmo el presente documento en el lugar y fecha que se indican a continuación.

Valencia, _______ de ____________ de 20__.

Nombre y apellidos del / de la participante:<br><br>Firma:	*Nombre y apellidos del padre, madre o tutor (en el caso de menores o incapaces)*:<br><br>Firma:	*Nombre y apellidos del investigador principal*:<br><br>Firma:

Si el sujeto del estudio es un adolescente capaz intelectual y emocionalmente de entre 12 y 16 años debe de ser oída su opinión y autorizar su participación en el estudio firmando también este consentimiento. Cuando se trate de menores no incapaces ni incapacitados, pero emancipados o con 16 años cumplidos, no cabe prestar el consentimiento por representación y será el propio sujeto del estudio quien firmará el consentimiento (Ley 41/2002).

REVOCACIÓN DEL CONSENTIMIENTO

Revoco el consentimiento prestado en fecha ______________________ para participar en el proyecto titulado "EFECTOS DE LA REHABILITACIÓN DOMICILIARIA DEL EQUILIBRIO BASADO EN EJERCICIOS FUNCIONALES EN PERSONAS CON ENFERMEDAD DE PARKINSON: ENSAYO CLÍNICO ALEATORIZADO" y, para que así conste, firmo la presente revocación.

En Valencia, a _______ de ____________________ de 20___.

Nombre y apellidos del / de la participante:<br><br>Firma:	*Nombre y apellidos del padre, madre o tutor (en el caso de menores o incapaces)*:<br><br>Firma:	*Nombre y apellidos del investigador principal*:<br><br>Firma:

CONSENTIMIENTO PARA LA TOMA DE IMÁGENES Y AUTORIZACIÓN PARA SU USO

Nombre de la persona: ____

Teléfono: ___________ Dirección: ______________________________

Nombre del padre, madre o tutor/a: ____

CONSENTIMIENTO PARA LA TOMA DE IMÁGENES

Por la presente, doy mi consentimiento para que se me tomen fotografías. El término “imagen” incluye video o fotografía fija, en formato digital o de otro tipo, y cualquier otro medio de registro o reproducción de imágenes.

Por la presente, autorizo el uso con fines didácticos o educativos.

PROPÓSITO

Por la presente, autorizo el uso de la(s) imágenes(s) para el propósito de difusión al personal del hospital, médicos, profesionales de la salud y miembros del público con fines educativos, de tratamiento, de investigación y científicos.

Doy mi consentimiento para que se tomen imágenes de mi hijo/a o tutorizado/a y autorizo el uso o la divulgación de tal(es) fotografía(s) a fin de contribuir con los objetivos científicos, de tratamiento, educativos, y por la presente renuncio a cualquier derecho a recibir compensación por tales usos en virtud de la autorización precedente. Por la presente, yo y mis sucesores o cesionarios eximimos al centro y a sus empleados, a mi(s) médico(s) y a cualquier otra persona que participe en mi atención, y a sus sucesores y cesionarios, de toda responsabilidad ante cualquier reclamo por daños o de indemnización que surja de las actividades autorizadas por este acuerdo.

REESCISIÓN

Si yo decido rescindir esta autorización, no se permitirá posteriores usos de mi fotografía o la de mi hijo/a, tutorizado/a, pero no podrá pedir que se devuelvan las fotografías o la información ya utilizadas.

DERECHOS

Puedo solicitar que cese la filmación o grabación en cualquier momento.

Puedo rescindir esta autorización hasta una fecha razonable antes de que se utilice la imagen, pero debo hacerlo por escrito, remitido a ____

Puedo inspeccionar u obtener una copia de las imágenes cuyo uso estoy autorizando.

Puedo negarme a firmar esta autorización. Mi negativa no afectará a las posibilidades de mi hijo de recibir atención.

Tengo derecho a recibir una copia de esta autorización.

Entiendo que no recibiré ningún tipo de compensación financiera.

FIRMA

Fecha: ______________________________

Firma: _________________ Firma: _________________ Firma: _________________

Paciente si es mayor de 12 años *representante legal* *Investigador prinicpal*

FIRMA REESCISIÓN

Fecha: ______________________________

Firma: _________________ Firma: _________________ Firma: _________________

Paciente si es mayor de 12 años *representante legal* *Investigador principal*
