# Supplementary material for: Functional balance training in people with Parkinson’s disease: a protocol of balanceHOME randomized control trial with crossover
Source: Front Aging Neurosci. 2023 Apr 26;15:1137360. doi: 10.3389/fnagi.2023.1137360 (PMC10231658; doi:10.3389/fnagi.2023.1137360)
Supplement: Supplementary file 4 [file Data_Sheet_4.pdf]

Standardized neurological clinical interview for Parkinson's disease

Personal data: **Code:**

Birthdate:	Sex: ☐ Woman ☐ Man
Dominance: ☐ Right-handed ☐ Left-handed<br>☐Ambidextrous	Civil status: ☐Single ☐Married ☐Couple<br>☐Separated ☐Divorced ☐Widowed
Lives with: ☐Partner ☐Children ☐Partner and child/ren ☐Alone ☐Others	Academic level:<br>☐ No studies ☐ Primary<br>☐ Secondary ☐ High school<br>☐Professional training ☐University
Current occupation:<br>☐Professional ☐Retired by age<br>☐Trade job ☐Retired due to illness<br>☐Work at home ☐Unemployed	Specify formation:<br>Specify year of retirement (if applicable):
Specify type of current or previously performed work (and number of years):	
Smoking habit:<br>☐ Do smoke, daily<br>☐ Do smoke, not daily<br>☐ Does not currently smoke, but has smoked before<br>☐ Does not smoke, nor has ever smoked regularly	Alcohol (specify amount):<br>☐ Every day<br>☐ Some day a week<br>☐ Some day a month<br>☐ Do not consume alcohol
Physical activity level: (specify activity and amount)<br>☐ Completely inactive (read, TV…)<br>☐ Occasional physical or sporting activity (walking, gardening, cycling, gymnastics…)<br>☐ Regular physical activity (walking, tennis, swimming…)	
Intellectual activity level: (specify activity and amount)<br>☐ Completely inactive<br>☐ Occasional intellectual activity<br>☐ Regular intellectual activity	
Has COVID-19 passed? (when, severity of symptoms, duration)<br>☐ Yes<br>☐No	
Have you been vaccinated against COVID-19? (when, vaccine)<br>☐ Yes<br>☐ No	

Parkinson start and history:

Year of diagnosis of Parkinson's disease:	Evolution in years to the current date:
Family history: ☐ no history ☐ mother ☐ father ☐ both parents ☐ sibling/s ☐ grandfather/s	
Other diagnoses (specify date):	
Parkinsonian medication, time taken and amount (mg):	Other medications, time taken and amount (mg):

Physiotherapy (where?): ☐ not performed ☐ 1 time/week ☐ 2 times/week ☐ 3 times/week ☐ 4 or more times/week	
Other therapies (which ones and frequency):	

Parkinsonian signs:

Hoehn and Yarh Stadium:	UPDRS III Score:
Predominance of signs: ☐Right hemibody ☐Left hemibody ☐Bilateral	
Side of the body where the signs first appear (specify):<br>☐ Right hemibody ☐ Left hemibody ☐ Both	
Rest tremor: ☐Right hemibody ☐Left hemibody ☐Both<br>☐Upper limb ☐Lower limb	
Bradykinesia: ☐Right hemibody ☐Left hemibody ☐Both<br>☐Upper limb ☐Lower limb	
Stiffness: ☐Right hemibody ☐Left hemibody ☐Both<br>☐Upper limb ☐Lower limb	
Postural alteration:<br>☐ Positive destabilization test ☐ Romberg + eyes open ☐ Romberg + eyes closed	
Other signs:	
MMII length: cm right cm left	
Number of falls in the last month:	

Comments/others:

____

____

____

____

____

____

____

Inclusion/exclusion criteria:

Inclusion criteria:	
PD diagnosis	☐ Yes ☐ No
H&Y Stadium between I and IV	☐ Yes ☐ No
Independent walking with or without technical assistance	☐ Yes ☐ No
Stable parkinsonian medication last month	☐ Yes ☐ No
Exclusion criteria:	
Presence of other diseases that affect balance or gait	☐ Yes ☐ No
Other diagnosed neurological pathologies	☐ Yes ☐ No
Uncontrolled chronic diseases	☐ Yes ☐ No
Participate in another physical rehabilitation program during the intervention period or 1 month before starting it	☐ Yes ☐ No
Not mastering one of the two official languages of the Valencian Community	☐ Yes ☐ No
